# Supplementary material for: Antennal Enriched Odorant Binding Proteins Are Required for Odor Communication in Glossina f. fuscipes
Source: Biomolecules. 2021 Apr 8;11(4):541. doi: 10.3390/biom11040541 (PMC8068202; doi:10.3390/biom11040541)
Supplement: Supplementary file 1 [file biomolecules-11-00541-s001.zip › Supplementary figures&tables.docx]

Supplementary figures and Tables

Supplementary figure 1:


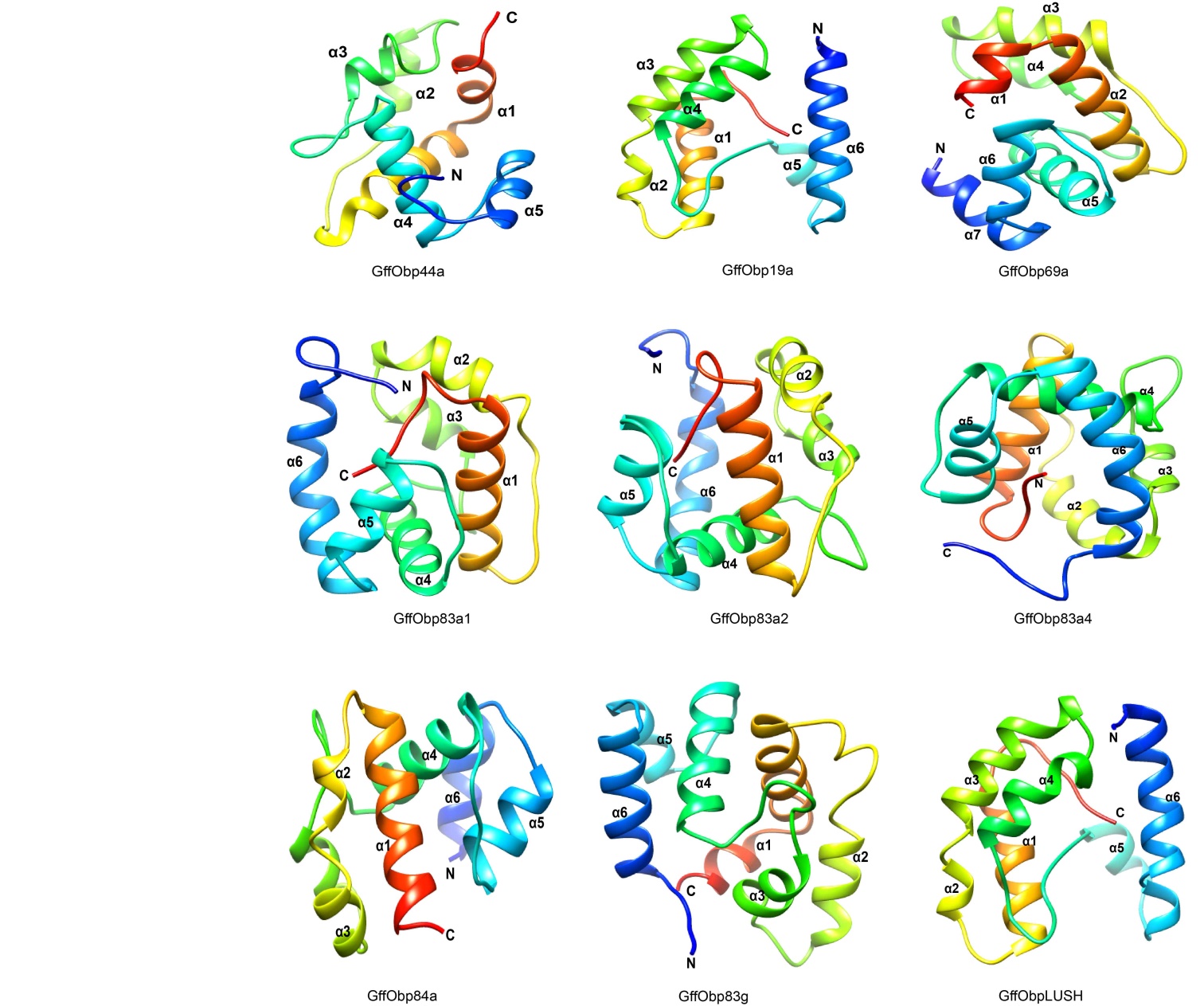


3D structure of 9 odorant binding proteins in *Glossina f. fuscipes* highlighting the number of α-helices.

Supplementary figure2


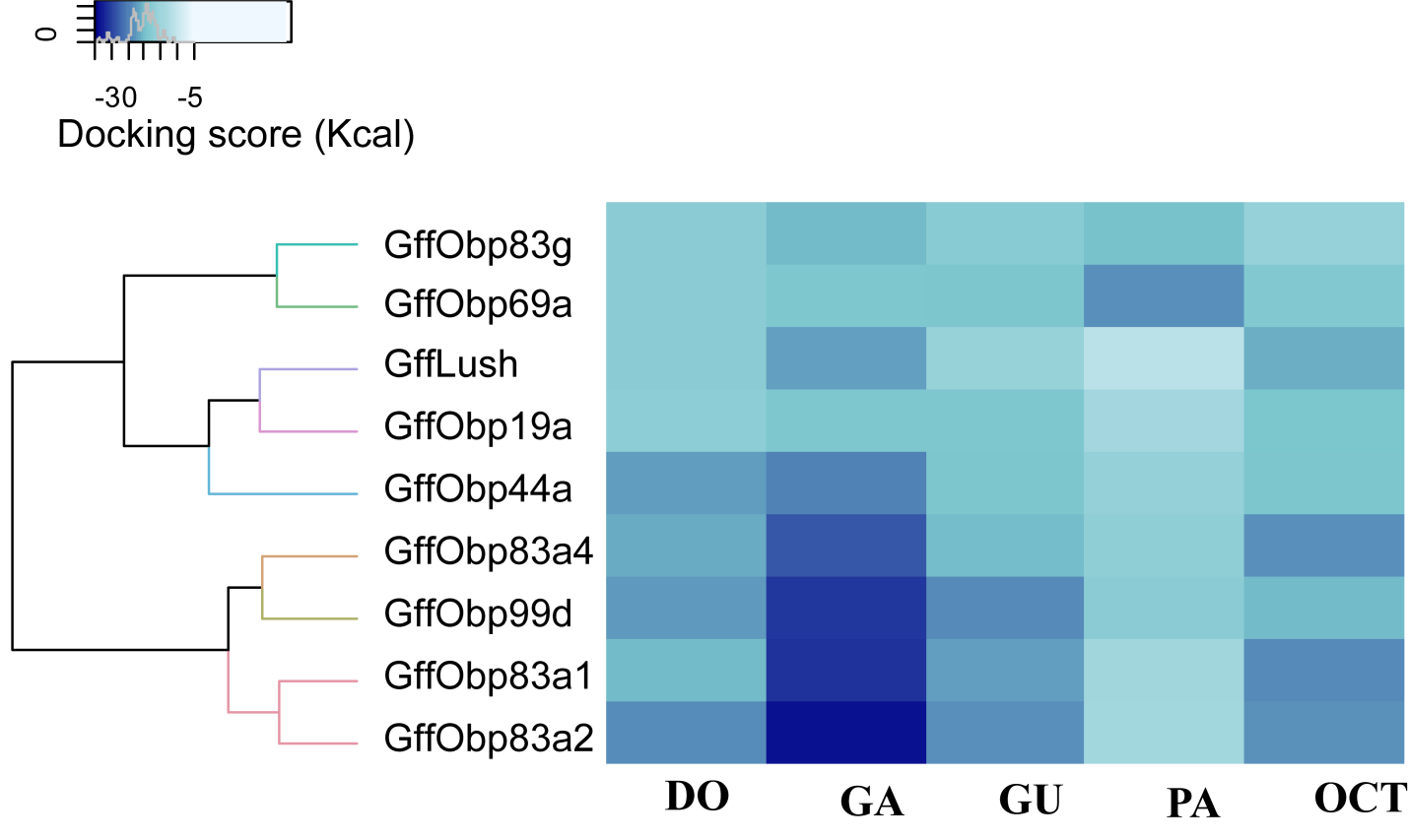


The molecular docking of different odorants binding proteins of *Glossina f. fuscipes*

Heatmap showing the binding affinity of different OBPs to Waterbuck repellent components and 1-octen-3-ol. DO= δ-octalctone; GA= geranylacetone; GU= guaiacol; PA= pentanoic acid; OCT= 1-octen-3-ol.

Supplementary figure 3


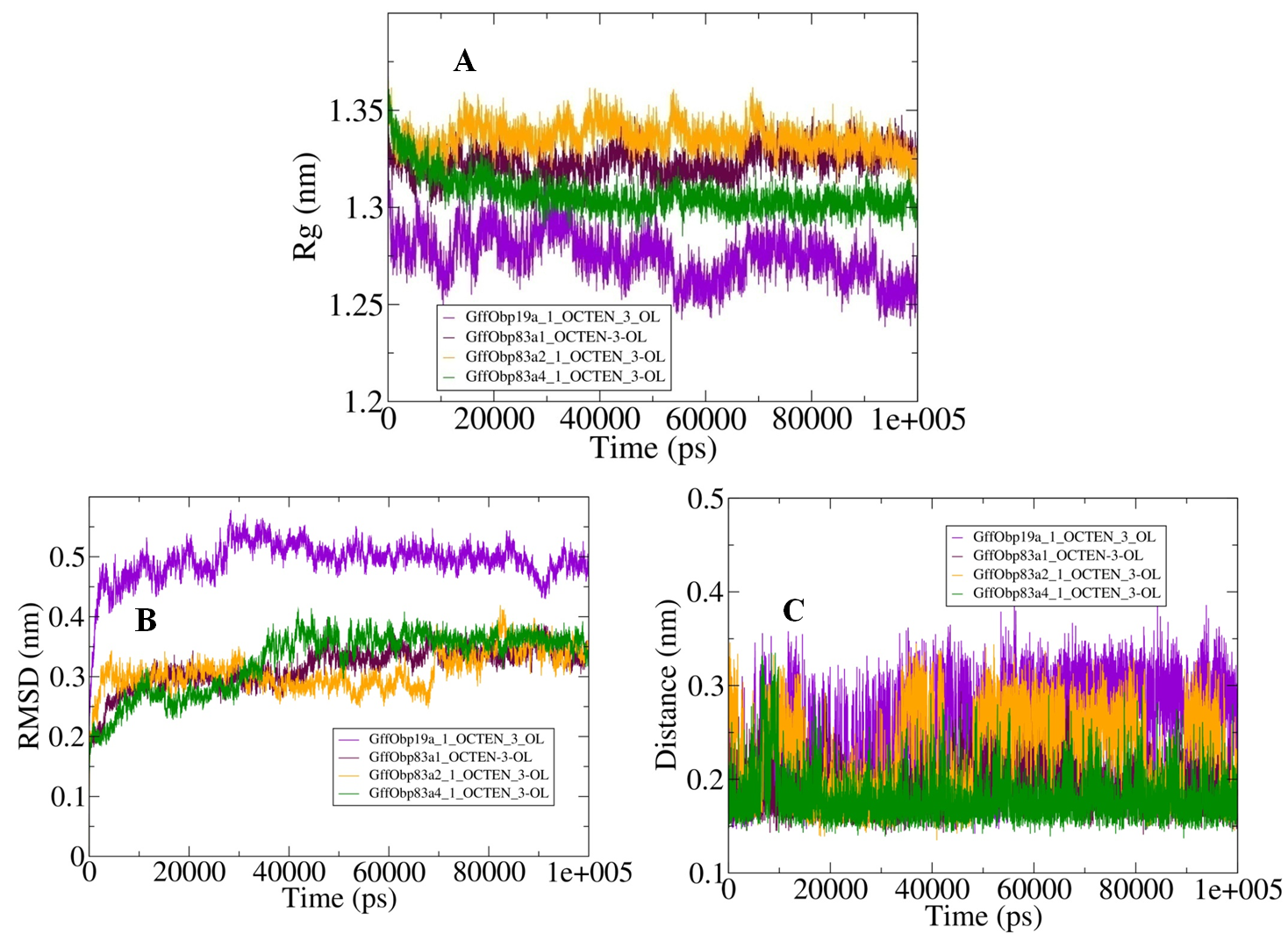


The MD simulations based parameters' curves (A) showing the changes observed in the compactness of the studied systems in terms of Radius of Gyration (Rg) (B) highlighting the changes observed in the Root Mean Square Deviation (RMSD) values (C
) illustrating the variation in the calculated distances between protein and ligands in the studied systems. (Purple - GffObp19a_1_OCTEN_3_OL, Maroon - GffObp83a1_OCTEN-3-OL, Orange - GffObp83a2_1_OCTEN_3-OL, Dark Green - GffObp83a4_1_OCTEN_3-OL)

Supplementary table 2. Summary of binding pocket dynamics and physicochemical characteristics of *Glossina f. fuscipes*

| Obp | Class of Obp | Volume (Å^3^) | Surface (Å^2^) | H bonds donors | H bonds acceptors | Hydrophobic interactions |
| --- | --- | --- | --- | --- | --- | --- |
| GffLush | Typical | 1025.28 | 1417.17 | 13 | 41 | 67 |
| GffObp69a | Typical | 1008.64 | 1416.96 | 19 | 46 | 89 |
| GffObp83a4 | Typical | 769.89 | 743.1 | 4 | 34 | 49 |
| GffObp83g | Typical | 922.69 | 1133.85 | 20 | 61 | 49 |
| GffObp99d | Typical | 1358.02 | 2058.23 | 31 | 74 | 96 |
| GffObp19a | Plus-C | 1426.18 | 2115.39 | 27 | 75 | 102 |
| GffObp83a1 | Plus-C | 935.1 | 1116.77 | 7 | 47 | 54 |
| GffObp83a2 | Plus-C | 1267.01 | 1558.91 | 12 | 59 | 87 |
| GffObp44a | C-Minus | 676.16 | 888.02 | 7 | 36 | 37 |

Supplementary table 3: Primers for RT-qPCR and RNAi experiments

| Name | Sequence |
| --- | --- |
| Gfflush_F | ATGTTAACTGCGTTAAAATG |
| Gfflush_R | GGACTCAGTCCCGACCCAAT |
| Gffobp19a_F | ATGTTTGGCAAAATATCGTA |
| Gffobp19a_R | AAGGGAAAACGAATTTTGTG |
| Gffobp83a1_F | ATGCTTTTAAAGTGTGATTG |
| Gffobp83a1_R | TCCCACACAAAACGATTATC |
| Gffobp83a2_F | GCAAGCAATGATTGTTAAGT |
| Gffobp83a2_R | CACTATGCACGCACTTGTCA |
| Gffobp83a4_F | AGGATGACTTTGTCCGGTAA |
| Gffobp83a4_R | TTGCAGTAGATGATCGATCT |
| T7Gfflush_F | TAATACGACTCACTATAGATGTTAACTGCGTTAAAATG |
| T7Gfflush_R | TAATACGACTCACTATAGGGACTCAGTCCCGACCCAAT |
| T7Gffobp19a_F | TAATACGACTCACTATAGATGTTTGGCAAAATATCGTA |
| T7Gffobp19a_R | TAATACGACTCACTATAGAAGGGAAAACGAATTTTGTG |
| T7Gffobp83a1_F | TAATACGACTCACTATAGATGCTTTTAAAGTGTGATTG |
| T7Gffobp83a1_R | TAATACGACTCACTATAGTCCCACACAAAACGATTATC |
| T7Gffobp83a2_F | TAATACGACTCACTATAGGCAAGCAATGATTGTTAAGT |
| T7Gffobp83a2_R | TAATACGACTCACTATAGCACTATGCACGCACTTGTCA |
| T7Gffobp83a4_F | TAATACGACTCACTATAGAGGATGACTTTGTCCGGTAA |
| T7Gffobp83a4_R | TAATACGACTCACTATAGTTGCAGTAGATGATCGATCT |

Supplementary table 4. Raw data generated from Behavioural assay of wild type gene knockdown flies.

| Species | Fly type | Replicate | Number release | Flies in Control | Flies in Treatment | No response | Dead flies | Attraction Index |
| --- | --- | --- | --- | --- | --- | --- | --- | --- |
| *Gff* | Obp83a1-/- | 1 | 20 | 7 | 3 | 10 | 0 | -0.173913043 |
| *Gff* | Obp83a1-/- | 2 | 20 | 2 | 5 | 11 | 2 | 0.15 |
| *Gff* | Obp83a1-/- | 3 | 20 | 8 | 0 | 12 | 0 | -0.363636364 |
| *Gff* | Obp83a2-/- | 1 | 20 | 2 | 1 | 15 | 2 | -0.055555556 |
| *Gff* | Obp83a2-/- | 2 | 20 | 11 | 2 | 6 | 1 | -0.5 |
| *Gff* | Obp83a2-/- | 3 | 20 | 10 | 3 | 4 | 3 | -0.388888889 |
| *Gff* | Obp83a4--/ | 1 | 20 | 7 | 1 | 9 | 3 | -0.315789474 |
| *Gff* | Obp83a4-/- | 2 | 20 | 5 | 6 | 7 | 2 | 0.05 |
| Gff | Obp83a4-/- | 3 | 20 | 8 | 1 | 9 | 2 | -0.388888889 |
| Gff | Op19a-/- | 1 | 20 | 0 | 10 | 10 | 0 | 0.5 |
| Gff | Op19a-/- | 2 | 20 | 1 | 12 | 7 | 0 | 0.47826087 |
| Gff | Op19a-/- | 3 | 20 | 1 | 16 | 3 | 0 | 0.681818182 |
| Gff | Wild type | 1 | 20 | 1 | 11 | 8 | 0 | 0.5 |
| Gff | Wild type | 2 | 20 | 4 | 13 | 3 | 0 | 0.45 |
| Gff | Wild type | 3 | 20 | 4 | 14 | 3 | 0 | 0.434782609 |
| Gff | Wild type | 4 | 20 | 3 | 15 | 2 | 0 | 0.6 |
| Gff | Wild type - NFW | 1 | 20 | 4 | 8 | 5 | 3 | 0.2 |
| Gff | Wild type - NFW | 2 | 20 | 2 | 14 | 2 | 2 | 0.6 |
| Gff | Wild type - NFW | 3 | 20 | 5 | 9 | 3 | 3 | 0.2 |
